# Morphological Sensitivity to pH of Silica and Chalk Nanocrystalline Self‐Organized Biomorphs

**DOI:** 10.1002/smsc.202400090

**Published:** 2024-06-11

**Authors:** Arianna Menichetti, Jeannette Manzi, Fermín Otálora, Marco Montalti, Juan Manuel García‐Ruiz

**Affiliations:** ^1^ Department of Chemistry “Giacomo Ciamician” University of Bologna Via Selmi 2 40126 Bologna Italy; ^2^ Laboratorio de Estudios Cristalográficos Instituto Andaluz de Ciencias de la Tierra CSIC‐Universidad de Granada Av. De las Palmeras 4 18100 Armilla Spain; ^3^ Donostia International Physics Center Paseo Manuel de Lardizabal nº 4 20018 Donostia‐San Sebastián, Gipuzkoa Spain

**Keywords:** biomorphs, counter‐diffusion, fractal growth, pH fluorescent probe, self‐organization, witherite

## Abstract

Inorganic–inorganic self‐organized composite architectures resulting from the chemical coupling of alkaline‐earth carbonate and polymeric silica are a promising alternative to organic‐based hybrid bio‐mimetic systems for developing innovative multi‐functional materials. Although the importance of pH in the generation of these structures reminiscent of primitive living organisms (and for this called biomorphs) is widely acknowledged, the effect of pH is generally investigated on the basis of starting pH value. This approach inadvertently neglects the important spatial and temporal pH gradients associated with biomorph nucleation and growth. A deep understanding of the role of pH on morphogenesis requires the ability to detect locally the pH in real‐time with a non‐invasive technique and correlate pH to the different stages of biomorphic growth. This aim is achieved by combining optical and fluorescence imaging. An accurately selected pH probe suitable for ratiometric pH measurement in the silica gel is exploited during a typical counter‐diffusion experiment. The results are compared with computer simulation of the synthesis of biomorphs by counter‐diffusion experiments. The results demonstrate the existence of two main morphogenetic regimes. Interestingly, the morphogenetic process controlling the complex shaping of biomorphs results to be independent of the silica speciation.

## Graphical abstract

Combining optical, fluorescence ratiometric pH imaging, and computer simulation during the synthesis of biomorphs by counter‐diffusion experiments the existence of two main morphogenetical regimes is demonstrated. Fractal growth occurs near the interface at pH values below 10. Biomorphic structures form within a narrow range of pH values, namely 10.3–10.4 independently of the distance from the interface.

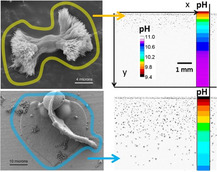

## 1 Introduction

**Figure 1:**
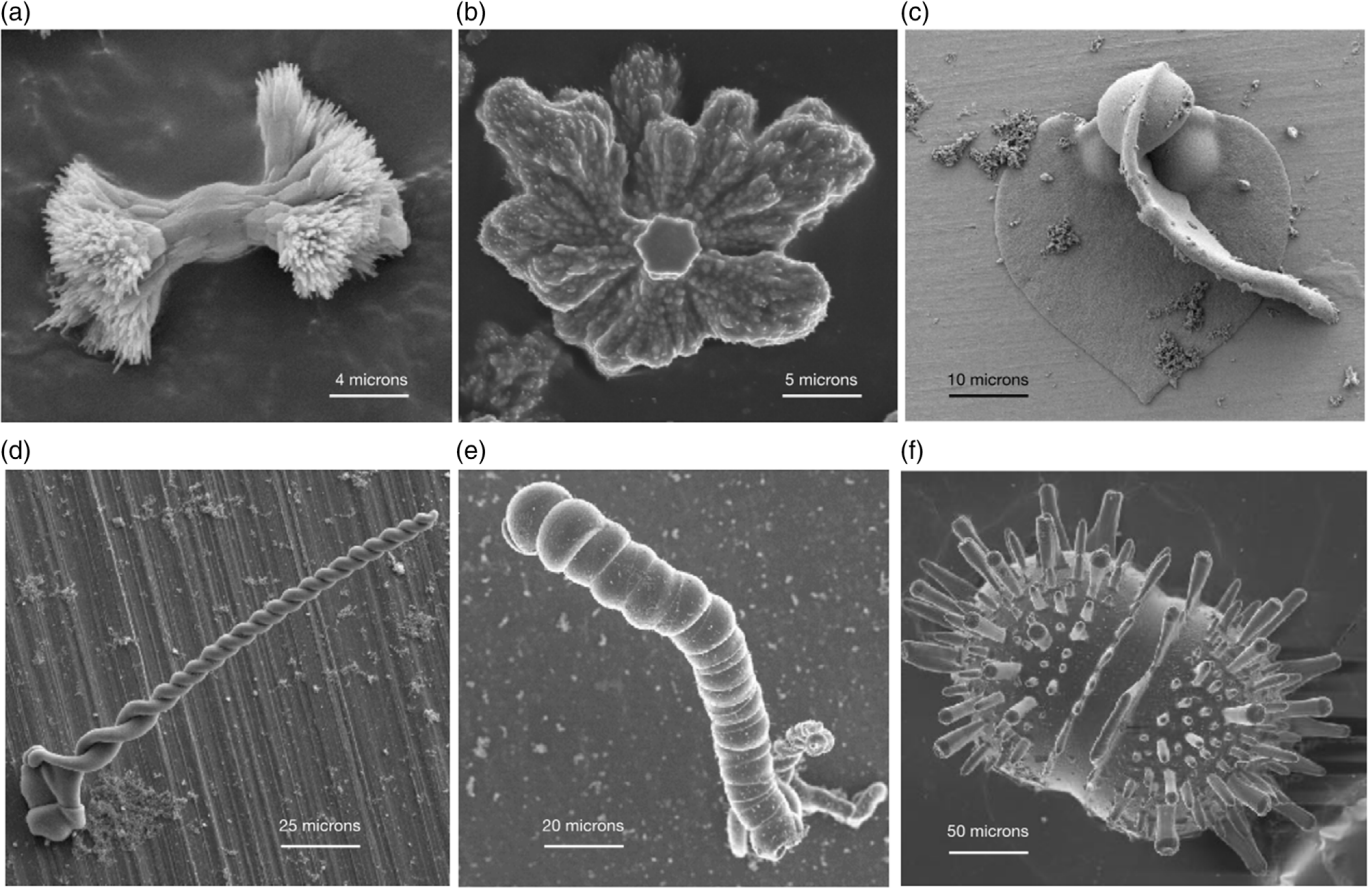
The morphology of carbonate‐silica biomorphs at two different regimes: a,b) Two stages of fractal growth; c–f) Biomorphic growth. c) cardioid‐like sheets; d) twisted ribbons, e) helicoids also known as “worms”; f) diplococci‐like biomorphs. a–e: Barium carbonate/silica biomorphs; f: Calcium carbonate/silica biomorph.

The bottom‐up design of hierarchically organized nanostructures is a powerful strategy to achieve new materials with innovative functionalities. To a large extent, this approach takes inspiration from the ability of living organisms to engineer hybrid nanocomposites both for structural and functional purposes. Intense research has been dedicated to synthesizing biologically inspired textures and shapes mimicking the hybrid route found by nature, a route based on organic polymers to guide nanocrystallization.^[^ ^1^, ^2^, ^3^ ^]^ An interesting alternative to the organic–inorganic hybrid structure is represented by the complex inorganic–inorganic architectures resulting from the chemical coupling of polymeric silica and carbonate.^[^ ^4^, ^5^, ^6^, ^7^, ^8^ ^]^ These structures are called “biomorphs” because their morphologies are reminiscent of the shape of primitive living organisms^[^ ^9^, ^10^, ^11^ ^]^ (**Figure** **1**). They are made of thousands of carbonate nanocrystals co‐oriented via silica‐induced interactions into various biomimetic structures that exhibit non‐crystallographic curved morphologies and hierarchical textures.^[^ ^6^, ^10^, ^12^, ^13^, ^14^, ^15^, ^16^ ^]^ The formation of biomorphs is thought to be an autocatalytic phenomenon triggered by the reverse pH dependency of the solubility of alkaline‐earth carbonates and silica: as the carbonate crystals form, the pH decreases due to the continuous removal of carbonate groups, and this decreasing pH induces the precipitation of amorphous silica; in turn, the precipitation of silica causes an increase in pH and therefore triggers a new event of carbonate nucleation, thus maintaining this autocatalytic cycle of coprecipitation.^[^ ^6^, ^17^ ^]^ The mechanism by which this is connected to morphogenesis is still under discussion.^[^ ^18^, ^19^ ^]^ The possibility of controlling the growth of biomorphs, particularly their morphology and texture, has been reported by tuning local parameters like temperature,^[^ ^4^, ^20^ ^]^ pCO_2_,^[^ ^18^ ^]^ and pH.^[^ ^17^, ^21^, ^22^, ^23^ ^]^

The importance of pH in the generation of biomorphs is beyond any doubt. First, because the concentration of one of the reactants, the carbonate groups, strongly depends on pH. Therefore, two key parameters, namely the supersaturation value for BaCO_3_ and the concentration ratio Ba/CO_3_ depend on pH. Indeed, CO_2_, based on the pH of the medium, can be present as gas phase, composite carbonic acid, bicarbonate (hydrogen carbonate), and carbonate. Thus, depending on the pH, the equilibria of the different species change, influencing BaCO_3_ solubility and Ba/CO_3_ concentration ratio.

**Figure 2:**
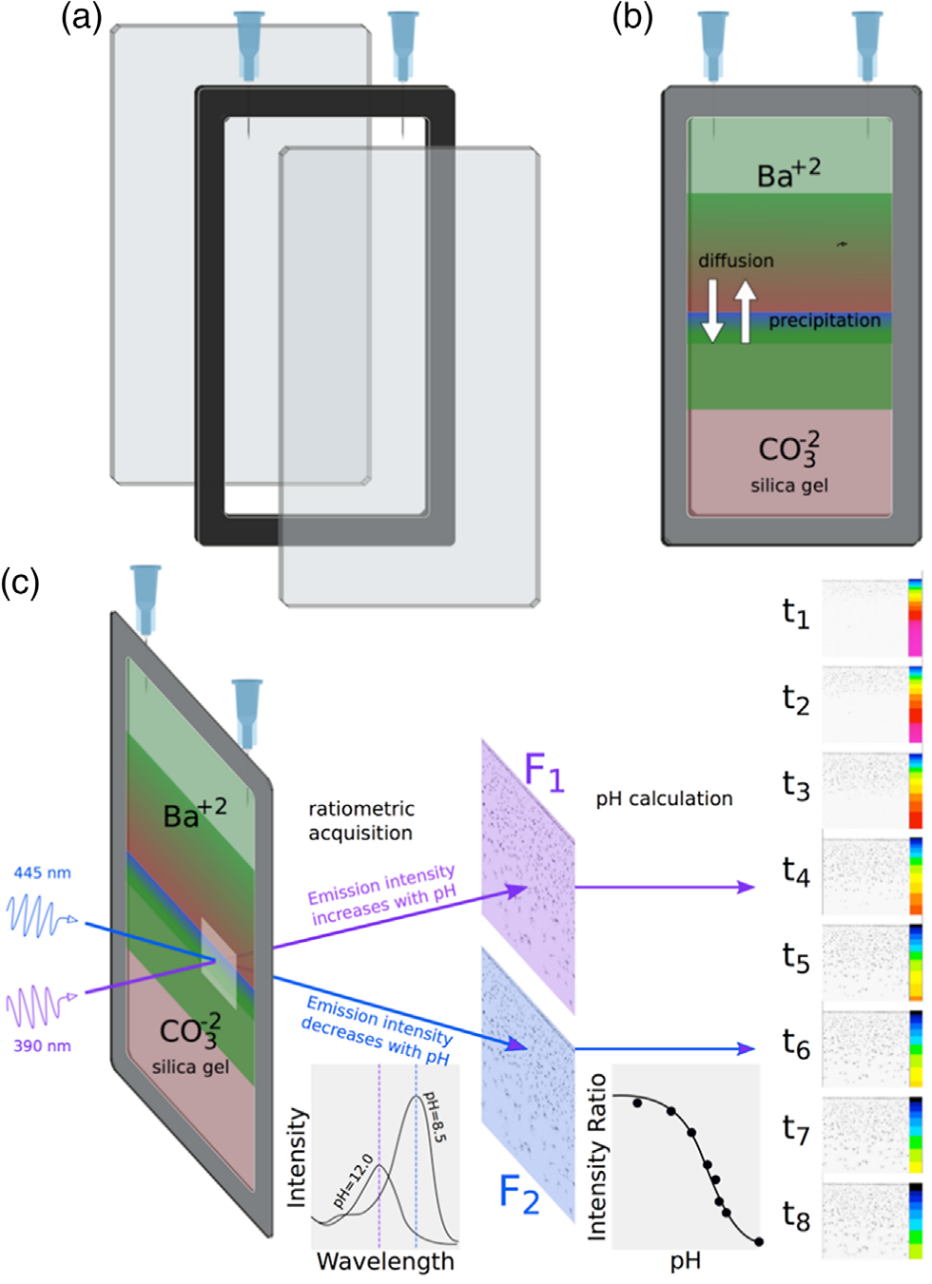
The gel counter‐diffusion technique for growing silica‐carbonate biomorphs. a,b) The assembly of the glass cassette; c) Sketch of the setup for ratiometric fluorescence signal acquisition during BaCl_2_ diffusion into the silica gel chamber.

Second, the speciation of silica, which plays a crucial role in morphogenesis, is highly dependent on pH. In the case of the counter‐diffusion method described in **Figure** **2**a–b, during the preparation of the cassettes, upon acidification, the silica gel forms by the condensation of molecules of silicic acid Si(OH)_4_. At alkaline pH, the silicic acid deprotonates, thus there are three solution species Si(OH)_4_, SiO(OH)_3_^−^, and SiO_2_(OH)_2_^2−^ that coexist in solution, in ratios controlled by pH. Thus, the physicochemical conditions, i.e., pH and species concentrations, change continuously for every *x*,*y* position in the gel, and each of them changes with time. However, so far, most studies have been based on correlating the biomorph morphology to the initial pH value of the silica solution or silica gel and the barium concentration.^[^ ^13^, ^24^ ^]^

A pioneering study of the spatial and temporal evolution of pH inside silica gels during the precipitation of witherite biomorphs was performed by Garcia‐Ruiz and coworkers.^[^ ^23^ ^]^ They correlated pH with a photographic record of the spatial and temporal evolution of the morphologies of the aggregates. Several problems were reported, namely: 1) the pH mapping was based on the gel sectioning and hence disruptive in that study; 2) a large gel volume was required for each pH measurement allowing a poor spatial resolution (>1 cm); and 3) The protocol requires the exposure to air, thus introducing unavoidable artifacts. The work demonstrated that a correct understanding of the role of pH on morphogenesis requires measuring the pH locally in real‐time with a non‐invasive technique. We recently demonstrated the use of fluorescence imaging to detect pH on the growing front of biomorph in solution, reporting the evidence of oscillations in the local pH at the front.^[^ ^17^ ^]^

Unfortunately, the same approach was demonstrated not to be suitable for mapping large (several centimeters) areas for long (several hours) times as required for mapping pH into the gel. To solve the problem, we have developed a ratiometric approach (Figure 2c), using a probe that can be excited at two different wavelengths (390 and 445 nm for the chosen probe—Figure S4, Supporting Information) to give two different fluorescence signals (Figure S5, Supporting Information), named *F*_1_ and *F*_2_, with different pH dependency (in particular, for the selected probe, *F*_1_ is almost pH independent). Advantageously, the ratio between the two signals *R* = *F*_2_/*F*_1_ is independent of the local concentration of the probe and can be used to calculate pH (Figure S6, Supporting Information). This methodology ruled out artifacts in the pH measurements due to probe concentration inhomogeneity caused by diffusion transport photobleaching, or chemical degradation in harsh alkaline conditions of the silica gel.^[^ ^17^, ^25^, ^26^ ^]^ In this article, we demonstrate the use of the ratiometric fluorescent pH chemosensor to measure the pH variation in time and space across the silica gel during the growth of barium carbonate biomorphs. Changes in the local pH were correlated to nucleation and biomorphic growth by combining optical transmission to fluorescence imaging.

## 2 Experimental Section

### 2.1 Experimental Characterization of Witherite Growth and Solution pH

A typical counter‐diffusion setup (Figure 2a,b) was adopted for biomorphs grown in silica gel. The reactor was made of two glass plates (120 × 70 mm) separated by a rubber spacer 1 mm thick to produce an empty rectangular cell of 100 × 50 × 1 mm in size. Optical imaging and local pH detection in real‐time in a non‐invasive way was achieved by a combination of optical and fluorescence imaging exploiting an accurately selected pH probe suitable for ratiometric detection in the silica gel, acridine yellow (AY) (Figure 2c). A probe that can be excited at two different wavelengths (390 and 445 nm for the chosen probe) was used to give two different fluorescence signals (*F*_1_ and *F*_2_) with different pH dependencies. A critical point in developing this approach was to consider the effect of the silica matrix on the response of the pH probe that depends on the pKa of the probe. For this, we prepared a set of gel samples at controlled pH incorporating the fluorescent probe to correlate the measured ratiometric signal to pH. We observed a large shift of Ka going from water to the silica matrix. The pH measured after gelling with a pH‐meter for the identical duplicated gel used as reference was 10.78.

#### 2.1.1 Preparation of the Silica Gel

Silica gels were prepared starting with commercial sodium trisilicate solutions from Sigma‐Aldrich (~27% in SiO_2_ and 13–14% in NaOH, which means a [SiO_2_]/[NaOH] molar ratio of ~1.33). This commercial solution was diluted 1:10 in volume with ultrapure water (Millipore ultrafiltrated) and then acidified with HCl 1M (325 μL mL^−1^) to gel the solution. The initial pH was 11.83, and after the addition of HCl decreased to 10.78. AY was used with a final concentration of 1 × 10^−5^ m. Then BaCl_2_ solution 0.25 M (pH = 6.93) was injected on the top.

#### 2.1.2 Fluorescence Images Acquisition

An Olympus IX‐71 inverted microscope equipped with a 4X Olympus objective was used for imaging in wide‐field modality. A lens was used to reduce magnification to 1.65×. Excitation was performed using a Xenon lamp and two different combinations of excitation/dichroic filters to acquire sequentially two images suitable for ratiometric analysis. Fluorescence F1 excitation was performed with MF 390‐18 (center wavelength: 390 nm, FWHM = 18 nm) and MD 416 (reflection band: 360–407 nm, transmission band: 425–575 nm). For fluorescence F2 excitation was performed with MF 445‐45 (center wavelength: 445 nm, FWHM = 45 nm) and MD 480 (reflection band: 415–470 nm, transmission band: 490–720 nm). The same emission filter MF 510‐42 (center wavelength: 510 nm, FWHM = 42 nm) was used in both cases. Fluorescence images (512 × 512 pixels, 1.65× magnification) were acquired using an EMCCD camera Princeton PhotonMAX 512 and analyzed using ImageJ software package^[^ ^27^ ^]^ with a dedicated macro. pH images 512 × 512 were binned to give 256 × 256 images to reduce the standard deviation (ΔpH = 0.05).

#### 2.1.3 Transmission Images Analysis

In transmission images, crystals appear as dark shapes against a bright background. Hence, in the digital images, they correspond to a minimum of intensity while the maximum identifies the background. To process the images sequence, the digital images were inverted, and the background was subtracted.

#### 2.1.4 Preparation of the Silica Gels for the Photophysical Characterization

Silica gels for photophysical characterization were prepared as described before (preparation of the silica gel section). A 3.0 mM water solution of AY was added to reach a final concentration of 30 μM. Then, the sodium silicate solution was portioned into a PMMA cuvette for fluorescence measurements (10 mm optical path, 3.5 mL maximum volume), dropping 2.5 mL of solution in each cuvette. Controlled amounts of an HCl solution 1.0 m were added to decrease the pH and trigger gelling. The cuvettes were stored for 5 days in the dark at room temperature. The pH of the cuvettes was measured with a digital pH meter equipped with a suitable electrode.

#### 2.1.5 Calibration of the Ratiometric Signal

After photophysical characterization of the AY in the silicate gel in the cuvette set described in the previous section, the gel was transferred into homemade glass cells obtained by spacing two microscope glass slides (25 × 75 mm) with a double‐sided adhesive tape (1 mm thick). A portion of the gel (150–200 mg) was positioned in the center of one of the two glasses before the cell was closed, giving a gel sample with a thickness of 1 mm and a surface of 1–1.5 cm^2^. The slide was then observed at the microscope, and the two fluorescence signals *F*_1_ and *F*_2_ were measured for the different samples having different pH. The ratio of the two signals *R* = *F*_2_/*F*_1_ was plotted as a pH function and fitted according to the model function.

(1) $$R=R_{1}+\left(R_{2}-R_{1}\right)/\left(1+10^{\text{pH}-{\text{pK}}_{a}}\right)$$

In particular, in this function:

(2) $$\text{at alkaline pH}\;(\text{pH}\gg \text{pKa})\;R=R_{1}$$

(3) $$\text{at acidic pH}\;(\text{pH}\ll \text{pKa})\;R=R_{2}$$

### 2.2 Computer Simulation

Counter diffusion precipitation of witherite was simulated using an ad‐hoc Python script implementing 1) one‐dimensional diffusive mass transport; 2) chemical speciation, via calls to the routines in the IPhreeqc modules;^[^ ^28^ ^]^ 3) nucleation kinetics; and 4) crystal growth kinetics. The script simulates the evolution of a 5 cm uni‐dimensional space initially filled with a 0.25 molal solution of BaCl_2_ (for 0 < *x* < 2.5 cm) and a 3.5 milimolal solution of Na_2_CO_3_ (for 2.5 < *x* < 5.0 cm). The composition of each discretized volume (100 segments separated by Δ*x *= 0.5 mm) was stored and used to compute the appropriate input to the hydrochemical code Phreeqc version 3.6.2 (database phreeqc.dat). In this step, activity coefficients were computed using Debye–Hückel's theory, and the equilibrium concentration of each solution species was computed. Diffusive mass transport was also computed by Phreeqc using time steps Δ*t *= 60 s, closed boundaries at both ends of the 1D space, and a diffusion coefficient *D* = 0.25e−9 for the ionic species. Saturation indices (logarithm of the ionic activity product divided by the equilibrium constant for the dissolution equation) were also computed during the Phreeqc for all the solid phases that can eventually precipitate from the mix of solution species. After each transport and speciation step, the concentration of each of the solution species and the saturation index for each phase were used to update the simulation's status and compute the amount of solid phase precipitated. This step was implemented as functions within our Python script to allow for using different kinetics for nucleation and growth. Nucleation kinetics were modeled by a sigmoidal function *n* = 1 − 1/(1 + exp((*s* − 3)/0.1)), where *n* is the amount (in mole) of witherite precipitating, and s is the saturation index. The s−3 term implies that the maximum nucleation rate occurs at a supersaturation 1e3. Growth and dissolution kinetics were also modeled by a sigmoidal curve *g* = 1 − 2/(1 + exp((s))) that saturates at higher s values, modeling the transition from kinetically controlled growth to diffusion‐limited growth. For each of the 100 discrete cells, the state of the solution was recorded every 10 min (10 iterations) in a data file containing values for the position, time, pH, total concentration of C, Ba, Cl, and Na, concentration, and activity of all aqueous species, witherite saturation index and precipitated amount. This data file was used for the analysis and plots.

## 3 Results and Discussion

### 3.1 Counter Diffusion Experiments

We have used the two‐layer counter‐diffusion method for gel growth.^[^ ^10^ ^]^ It consists of arranging two reactants, barium and carbonate, one on top of the other and allowing them to counter‐diffuse. The experiments are typically performed within a glass cassette in which a silica solution is allowed to gel at the desired alkaline pH (Figure 2). This solution is equilibrated with atmospheric CO_2_, thus providing the carbonate reactant. Upon gelling, an aqueous solution of the metal reactant (barium) is injected on top of the gel. Provided the concentration of the barium solution is higher than the concentration of carbonate, the precipitation front of the forming metal carbonate will move across the silica gel. The characteristics of gel growth are: 1) The synthesis occurs in a diffusive mass transport environment; 2) biomorphs nucleate and grow in the same location; 3) there is a gradient of growth conditions across the gel; and 4) this gradient is expressed in a morphological and textural evolution as clearly analyzed by Bittarello and Aquilano (2007),^[^ ^29^ ^]^ reporting a fractal growth followed by a biomorphic growth across the gel. Compared to solution growth, such as the one reported in the paper of Knoll et al.^[^ ^30^ ^]^ in which the front growth of a specific biomorph morphology (cardioid sheet) is completely monitored and characterized, biomorphs forming in counter‐diffusion gel experiments are more difficult to harvest for further ex‐situ characterization studies, but the gel technique provides an excellent medium for understanding morphogenesis.

The in‐situ experimental measurement of the local variation of pH in time and space during biomorphs nucleation and growth was investigated in a counter‐diffusion reactor (Figure 2). The cell was half‐filled with the silica sol containing a ratiometric pH fluorescent probe, AY. In particular, the ratio (*R* = *F*_2_/*F*_1_) between the fluorescence of the protonated form (excitation at 450 nm) and of the de‐protonated form (excitation at 390 nm) of AY, are correlated to pH and can be used to calculate it. Indeed, fluorescence signals, subjected to the concentration of the probe, can be affected by diffusion, photobleaching, or chemical degradation in the harsh alkaline conditions of the silica gel upon prolonged (several days) analysis. This would provide artifacts in the measurement of the local pH. At the contrary, the ratiometric signal, based on the ratio between the two fluorescence signals, does not depend on the local concentration of the probe, eliminating any artifact and providing a reliable correlation of the signal to the pH. This ratiometric approach widely exploited for investigating biological samples was very rarely applied to the inorganic system and here it was adapted for the first time to work in the alkaline pH window where biomorphs are formed. Further details on the experimental procedures and the acquisition and processing of optical and fluorescence images are provided in the Supporting Information. The crystallization experiment was started by injecting a solution of BaCl_2_ 0.25 m in the empty part of the cassette. During the counter diffusion experiment, fluorescence images of the silica gel at 510 nm were acquired.

**Figure 3:**
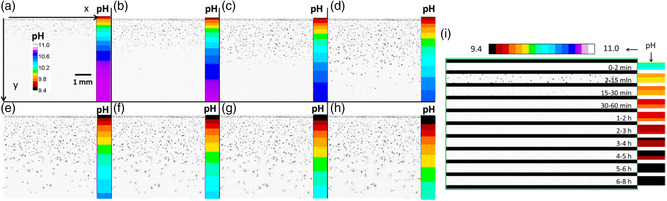
a–h) Optical transmission images of the silica gel in the cassette and local pH measure by fluorescence after the injection of the BaCl_2_ solution into the crystallization cassette: a) after 15 min, b) after 30 min, c) after 1 h, d) after 2 h, e) after 3 h, f) after 4 h, g) after 6 h, h) after 8 h. i). Optical transmission image differences showing the changes in the optical transmission in a given time interval in the gel band close to the solution–gel interface correlated to the corresponding pH profiles.

**Figure 4:**
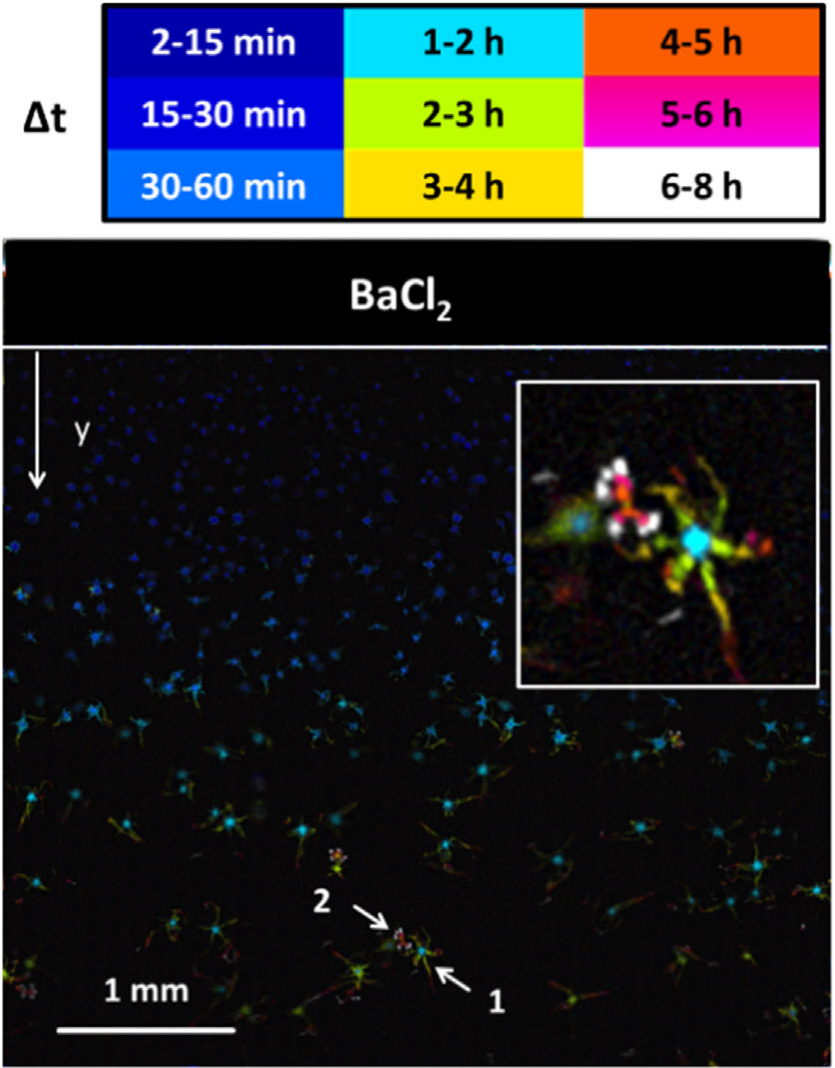
Optical transmission image differences show the changes in the optical image in a given time interval in the gel.

**Figure 5:**
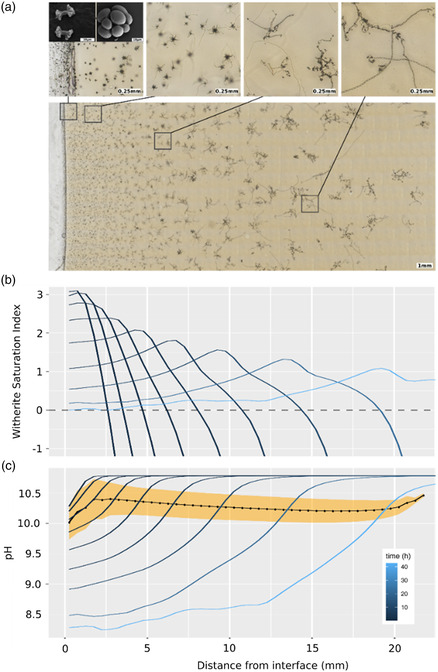
a) Extended resolution and depth of focus picture composed from 35 (*x*) times 8 (*y*) times 50 (*z*) images collected from a cassette containing gelled solutions. The grid of 35 × 8 images was collected at a grid of *x,y* positions using an automated motorized stage in a Nikon Eclipse Ti microscope. At each of these 280 positions, a stack of 50 images was collected by shifting the z position of the 10× objective to collect in‐focus images for every feature within 0.5 mm of the sample. Focus stacks and stitched images were computed using the enblend/enfuse software (http://enblend.sourceforge.net/) that created this extended resolution and depth of focus picture out of the 14 000 individual pictures. Inset at top/left: SEM images of fractal dendrites and globular structures. b) Evolution of the concentration of the witherite saturation index; c) evolution of the pH inside the chamber. Each curve represents the status of the gel chamber at a time 10, 20, 40,…,1280 min (0.17–21.33 h in log2 scale). The black dots and the line connecting them show the mean pH at which witherite precipitates at the corresponding position. The yellow band spans twice the standard derivation of the pH values (values at different times for the given position) centered at the mean pH.

Fluorescence images were acquired first upon excitation at 390 nm (image *F*_1_) and then upon excitation at 445 nm (image *F*_2_) at different delays time t_i_ with respect to the time of injection of the BaCl_2_ solution. The two images were analyzed to calculate the pH value as a function of the coordinates *x* and *y*, where *x* is the direction parallel to the solution–gel interface and *y* is the direction perpendicular to *x* and oriented from the BaCl_2_ solution toward the gel, as shown in **Figure** **3**a. Image J^[^ ^28^ ^]^ was used to calculate the image ratio *R* = *F*_2_/*F*_1_ that was hence converted into pH according to the calibration curve shown in Figure S6, Supporting Information. Optical transmission images of the biomorphs forming in the gel were also acquired in the same experiment and, since the pH distribution along the *x*‐axis was, as expected for the counter‐diffusion experiment, found to be narrow (<0.1 pH units), pH was observed to keep constant across this axis and just dependent on the distance from the interface as assumed for the model described in Figure 2b. Figure 3a–h show the transmission images of the reactor together with the respective pH local values (measured by fluorescence and shown in false colors on the right) at different times after BaCl_2_ injection. These transmission images allow to identify the position of the precipitation front at different times and to compare the pH at that specific distance from the interface to the predicted values of the computer simulation of the process reported below (Figure 5b–c). In Figure 3a, acquired 15 min after the beginning of the experiment, the crystallization front is at about 1.0 mm from the interface, along *y*‐axis, and the local pH is 10.3, in good agreement with the value predicted in the computational model Figure 5c. In Figure 3c–d the crystallization front moves at increasing distance from the interface and the local pH is about 10.4, because of witherite precipitation, and as expected from the computational model described below (Figure 5c); this pH variation has no effect on the morphology of the resulting biomorphs. Notice that transmission images demonstrate that the crystals formed at distances from the solution‐gel front larger than 0.5 mm finally evolve into biomorphic structures. Figure 3a–h also show that after crystallization starts, the growth of the biomorphic structures is associated with a local pH decrease, as expected from the predicted pH profiles of Figure 5c. In order to analyze more in detail the behavior of the system at the beginning of the diffusion we focused on the 0.5 mm gel band near the liquid–gel interface. In particular, to analyze the correlation between biomorphic growth and pH more in detail, we calculated the transmission image changes in time Δ Im (*t*_*i*_). We processed the transmission images sequence (time‐lapsed acquisition) by calculating the difference between two consecutive images. Hence considering the sequence as Im (t_0_), Im (*t*_1_), …, Im (*t*_*n*_) where *t*_*i*_ is the time at which the image has been acquired (after the injection of the BaCl_2_ solution), we calculated the transmission images difference Δ Im (*t*_i_) = Im (*t*_*i*_) − Im (*t*_*i*−1_). Note that these image differences Δ Im (*t*_*i*_) show only the structures formed in the *t*_*i*_ − *t*_*i*−1_ time interval and can be used to identify the different stages of growth during time. The transmission image differences Δ Im (*t*_*i*_) for the silica gel band close to the interface (0 < *y* < 0.5 mm) are shown in Figure 3i, and they demonstrate that: 1) most crystals forming within the first 0.5 mm of the gel does in the 2–15 min time range; 2) these crystals show almost no growth after 15 min; 3) no new crystals are formed after the first 15 min of the experiment. As shown in Figure 3i, the time‐dependent changes of the optical transmission images, and hence the different stages of crystallization, can be associated with the local pH by using the fluorescence maps (on the right in Figure 3i). From these studies we achieve a new key result, namely 4) that the nucleation and growth of the crystals in the 0 < *y* < 0.5 mm occurs within the 10.1–9.8 pH range, in good agreement with the prediction of Figure 5c. Interestingly, this is the range of pH in which fractal forms prior the development of biomorphic structures have been demonstrated to occur.^[^ ^9^, ^31^, ^32^ ^]^ To correlate morphology, time‐evolution, position in the gel, and pH, the optical transmission data were processed to give the false‐color image of **Figure** **4**.

In this figure, each structure, or part of a structure, was represented with a color code corresponding to the time interval in which it was formed. For example, the biomorph labeled as 1 in Figure 4 started to grow after 1 h and continued to grow up to 4 h. This false‐color representation is compelling in showing the complicated correlation between crystal formation and growth with the position in the silica gel and time. In particular, the false‐color image in Figure 4 clearly shows that, in most cases, nucleation occurs at increasing time delays at increasing distances from the interface. In contrast, Figure 4 also shows the existence of second nucleation events, which are delayed with respect to the first nucleation at the same distance to the interface. It is the case of the crystal labeled as 2 in Figure 4: the color scale clearly shows that this structure is formed later than the biomorph 1, although the position along the *y*‐axis is the same. It is interesting to observe that the morphology of the final structure is very reminiscent of a fractal growth regime rather than that of a biomorphic one. pH maps were hence used to determine the pH characteristic of the second nucleation process. This analysis demonstrated that these structures are formed at a pH of about 10.1.

### 3.2 Computer Simulation

To better interpret the experimental results, we performed a computer simulation of the precipitation of barium carbonate (witherite) by counter‐diffusing barium chloride and sodium carbonate solutions at the initial pH and concentrations of the experiment, and for the geometry used in Figure 2. **Figure** **5**b shows the computed evolution of supersaturation in the gel layer. As the concentration of Ba decreases to the right and C concentration decreases to the left, there is a maximum in the ion activity product somewhere within the chamber. Figure S1, Supporting information shows the evolution of [Ba] and total carbon concentration [C] (including carbonate and bicarbonate anions) within the gel layer. As the concentration of Ba is much larger than that of CO_3_ concentration, the latter gets depleted during witherite precipitation, and C diffusion to the left is very small. Ba is in excess and is not depleted during precipitation; at a given point, as a consequence, diffusion of Ba to the right is only slightly modified by precipitation. This asymmetry has an impact on the distribution of reactants and, therefore, on the ion activity product. The net effect is that the supersaturation value of barium carbonate advances from the gel–solution interface across the gel as a wave with decreasing amplitude and increasing width (Figure 5b).^[^ ^33^ ^]^ This supersaturation wave controls the precipitation front, as shown in Figures S2 and S3, Supporting information. This is a behavior characteristic of counter‐diffusion crystallization, in which the whole transport‐reacting process is controlled by diffusion.^[^ ^34^ ^]^ Therefore, the precipitation front, which is defined by the loci of points at which, for the first time, the amplitude of the supersaturation wave reaches the critical value for nucleation, advances with a square root dependence on time, and the nucleation density decreases exponentially with the distance to the interface. This agrees with the observed precipitation pattern of the whole experiment displayed in Figure 5a. Note that biomorphic morphologies, i.e., cardioid‐like sheets, twisted ribbons, helicoids, “worms” and other morphologies with continuous curvature are observed across the whole gel, except for the first millimeters near the gel–solution interface and in the region of the solution closer to that interface (See Figure 1). The reason is the pH value at which precipitation takes place. The evolution of pH during the simulation is shown in Figure 5c. The diffusion of the acidic barium chloride solution accounts for the reduction of pH at the solution–gel interface from its initial value of 10.75. This leads to the development of a pH gradient advancing from left to right with time. The precipitation of witherite at the entry of the gel chamber keeps decreasing the pH to a value of around 8.5. The pH at the closed end of the chamber (right side of the plot) is constant at the initial value during the first 10 h. After this time, the closed boundary at the right part of the gel does not allow further diffusion, and the pH there falls below 10.75, quickly approaching values around 8.5 in the long‐term, homogeneous state. Both the predicted maximum and minimum pH values and the time evolution of the pH gradient are close to the experimentally observed gradient. However, the most relevant information is the pH value at which precipitation of barium carbonate occurs across the gel. This is shown by the black line and yellow band in Figure 5c. The black solid line with dots is the pH during precipitation at that point, defined as the mean of the pH at each point weighted by the rate of precipitation at that point. That is:

(4) $$\text{pH }\left(x,t\right)\cdot \frac{\delta W\left(x,t\right)}{W\left(x\right)}$$

where pH (*x, t*) is the pH at point *×* at time *t*, *δW* (*x*, *t*) is the amount of witherite precipitated at point *x* in the instant *t*, and *W* is the total of witherite precipitated at point *x* during the entire experiment. The summation of *δW* (*x*, *t*)/*W*(*x*) equals 1, and are the “weights” for the mean. A given pH contributes to the mean proportionally to the fraction of witherite that precipitated at that pH value. The pH values at which no witherite precipitated have a weight of 0. The yellow band is the mean pH region (defined earlier) plus/minus one standard deviation, i.e., is an interval of significance of the mean values of the dotted line. The main conclusion is that except for the region nearer the interface solution‐gel, the precipitation of witherite occurs at a constant pH of 10.3–10.4. Near the interface, the pH values at which precipitation occurs is lower. In this area, the pH value is affected by the diffusion of barium chloride solution of initial pH 6 and the fast precipitation of witherite at high supersaturation. Precipitation taking place in the solution side of the gel interface solution might have values closer to 9.5 while the precipitation in the few millimeters close to the interface occurs at values near 10. Interestingly, there is a significant difference in the morphological behavior of the structures forming at pH close to 10 and those forming at pH values 10.3–10.4. As shown in Figure 3a–i, at pH values 10 dumbbell shapes form that later evolve to fractal dendrites and globular structures, as also shown in Figure 5a. This morphological behavior is consistent with the reported morphogenesis of biomorphs.^[^ ^9^, ^32^ ^]^

## 4 Conclusion

We have measured in‐situ the variation of pH within a counter‐diffusion precipitation experiment forming barium carbonate biomorphs and compared it with a computer simulation of the growth experiment under identical starting conditions. The results demonstrate the envisaged existence of only two pH‐dependent regimes in the formation of biomorphs:^[^ ^9^ ^]^ 1) At the region closer to the gel–solution interface, fractal structures form at pH lower than 10.1, i.e., when H_4_SiO_4_^0^ species dominate H_3_SiO_4_^−^ species; and 2) All the biomorphic structures, such as leave‐like sheets, twisted ribbons helicoidal structures, and worms, formed at the same narrow range of pH values (10.3–10.4 for the starting conditions of our experiment), i.e., in the region where H_3_SiO_4_^−^ species dominate H_4_SiO_4_^0^ and H_2_SiO_4_^=^. It means that whereas there is a substantial effect of pH (and then of silica species) on the transition from fractal to biomorphic growth, the morphogenetic mechanism leading to different biomorphic shapes is independent of pH, and must be inherent to the propagation of the growth front.

## Conflict of Interest

The authors declare no conflict of interest.

## Supporting information

Supplementary Material

## Data Availability

The data that support the findings of this study are available in the supplementary material of this article.
