# Supplementary material for: Morphological Sensitivity to pH of Silica and Chalk Nanocrystalline Self‐Organized Biomorphs
Source: Small Sci. 2024 Jun 11;4(8):2400090. doi: 10.1002/smsc.202400090 (PMC11935136; doi:10.1002/smsc.202400090)
Supplement: Supplementary file 1 — Supplementary Material [file SMSC-4-2400090-s001.pdf]

Supporting Information for "Morphological sensitivity to pH of silica and chalk nanocrystalline self-organized biomorphs"

Arianna Menichetti, Jeannette Manzi, Fermín Otálora, Marco Montalti and Juan Manuel García-Ruiz

Computer simulation of witherite precipitation in counter diffusion

Additional plots showing the simulation output are presented below.

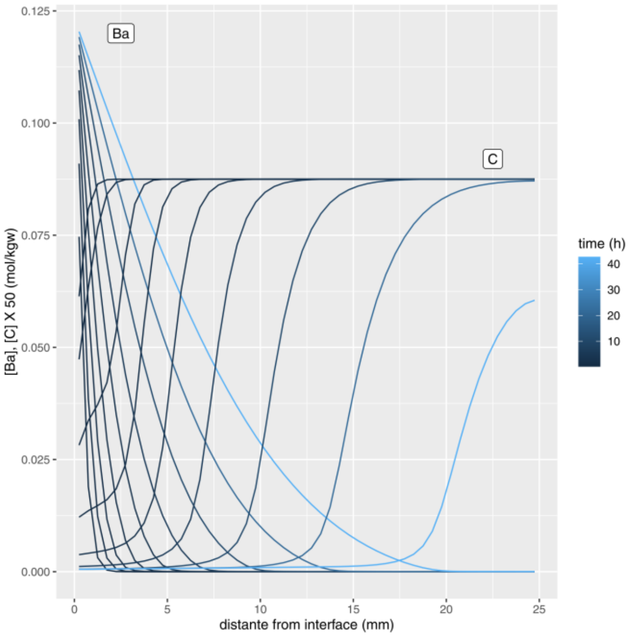

Figure S1. Evolution of [Ba] and total carbon concentration [C] (including carbonate and bicarbonate anions) within the gel layer. The curves for [Ba] show a typically diffusive (sigmoidal) shape, while the [C] curves (scaled x50 for clarity in the plot) show important deviations for the low values. This shows the role of "limiting reactant" played by carbonate: a significant part of the available carbonate (and only a small fraction of the available Ba) is consumed by the precipitation of witherite, especially in the early stages, when the precipitation is more active.

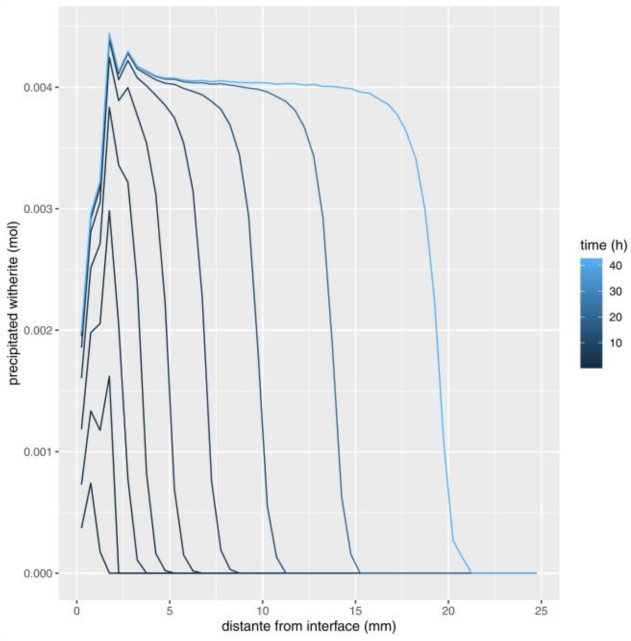

Figure S2. Evolution of the amount of witherite precipitated along the growth chamber during the simulation. The curves show an initial noise stage leading to a banded precipitation, followed by a stable precipitation advancing as a precipitation front and reaching a constant average amount of around 4mM of witherite in each of the 0.5mm slots simulated.

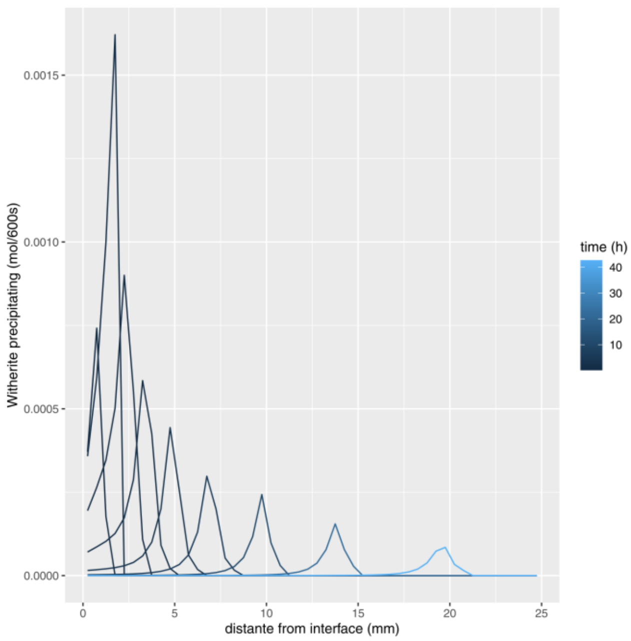

Figure S3. Evolution of the amount of witherite precipitated in each of the simulated slots during the discrete time interval Δt=60s. This plot show the precipitation front of decreasing amplitude and increasing width.

Testing of the pH probe in silica gel

Acridine yellow in the protonated form (AYH^+) undergoes the equilibrium:

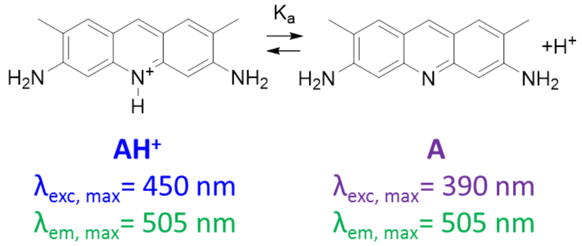

Figure S4. Equilibrium of protonation of the fluorescent pH probe acridine yellow (AY).

with an acidic constant pK_a= 8.9 in ultrapure water[1]. To evaluate the effect of pH on the fluorescence of AY in the silica gel, we prepared a set of gel samples containing AY at concentration 30 µM with a pH varying between 8.5 and 12.0. The pH was measured with a pH meter, and the fluorescence spectra were acquired upon excitation at λ_{exc1}=390 nm and λ_{exc2}=445 nm, and the excitation spectra upon λ_{em}=510 nm.

Figure S5 shows the emission and excitation spectra for the sample at pH 8.5 and 12. The emission spectra show similar profiles and peak wavelength and excitation wavelength but different intensities. In particular, the fluorescence intensity upon λ_{exc1}= 390 nm (blue lines in Figure S5) increases by about 40% going from pH 8.5 to pH 12 while the emission upon λ_{exc2}= 445 (red lines in Figure S5) is weak at pH 12 and it increases by roughly 20 times upon lowering pH to 8.5. Thanks to this different pH behavior, the two signals can be combined to measure the pH in a ratiometric approach. To identify the species responsible for the different emissions, the excitation spectra at λ_{em}= 510 nm at pH 8.5 and 12 (black lines in Figure S5) were compared with the absorption spectra of the neutral and protonated form of A in water (green lines in Figure S5; the non-optimal transparency of the gels precluded the measurement of its absorption spectra). The good marching of the spectra confirmed the formation of the protonated form AH^+ at pH=8.5 and of the neutral A at pH=12.

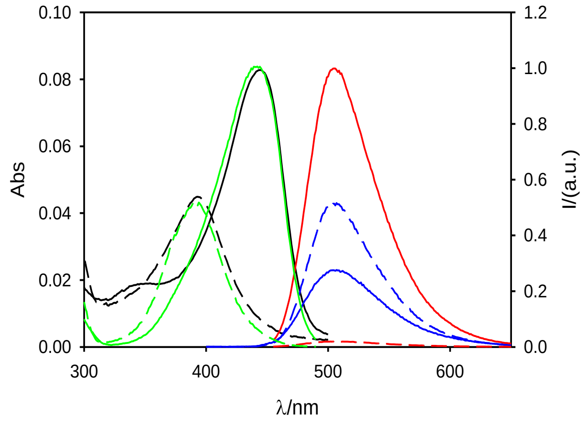

Figure S5. Fluorescence emission spectra of gels containing A (30 µM) upon excitation at λexc=390 nm (blue lines) and λexc=445 nm (red lines). Continuous lines are for the gel at pH 8.5, and dashed lines for the gel at pH 12.0 Excitation spectra at λem=510 nm are shown as black lines (continuous for pH=8.5 and dashed for pH 12.0. Excitation spectra are compared with the absorption of AH+ (solid green line) and A (dashed green line) in water.

To obtain a calibration curve, the fluorescence intensities of the gels at the two-excitation wavelength were performed with and epifluorescence microscopy on 1 mm thick samples of the silica gel. The fluorescence intensity F_1 (upon excitation at 390 ± 9 nm) and F_2 (upon excitation at 445 ± 22.5 nm) were used to calculate the intensity ratio $R=F_2/F_1$, which is independent of the concentration of the probe in the silica gel. The ratio R as a function of pH is shown at the left of Figure S6 (black squares), and it was fitted according to the equilibrium shown in Figure S4. This calibration curve was used to convert the fluorescence signal into pH in the counter diffusion experiment.

The excited-state lifetime (τ) and the fluorescence anisotropy (r) were also measured as a function of the pH, and they were reported in Figure S6 (right). The excited-state lifetime ranges between 4.0-4.5 ns, and it is, to a large extend, pH-independent as expected for the pH probe AY. The fluorescence anisotropy measures the rate of reorientation of the transition dipole after the excitation, and it can reflect a reduced rotation freedom. In the gel, r is rather pH-independent but also relatively high (r~0.25), revealing a partial interaction of the probe with the gel.

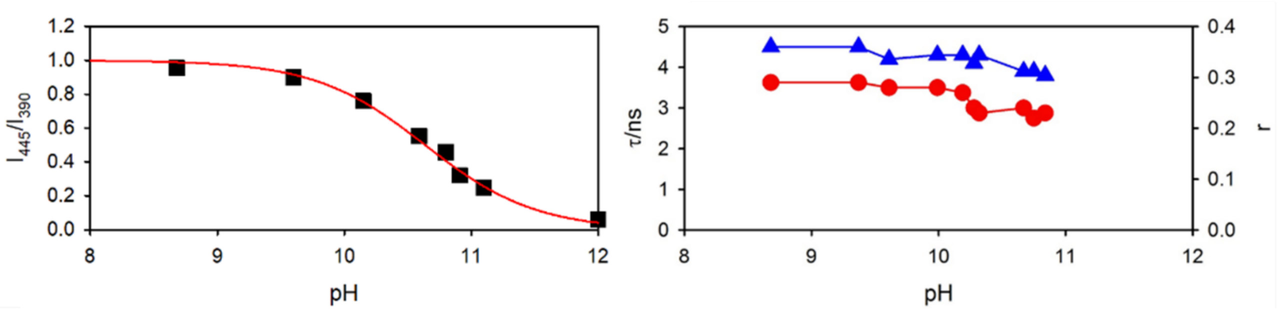

Figure S6. **Characterization of the ratiometric probe in the gel.** Left. Calibration curve of the ratiometric probe A in the gel. The ratio of the intensity of the fluorescence at 510 nm upon excitation at λ_{exc}=390 nm and λ_{exc}=445 nm (black squares) was measured with an inverted fluorescence microscope as a function of the gel pH and fitted according to the equilibrium of Figure S4. Right. The excited-state lifetime (blue triangles) and the fluorescence anisotropy (red circles) of A in the gel were measured as a function of pH.

Figure S7a shows the transmission image of the cassette two minutes after $BaCl_2$ injection together with the respective pH images shown in false colors in Figure S7b. Figure S7b clearly shows that a pH gradient is already present in the silica gel near the interface as soon as two minutes after the beginning of the experiment, i.e., after injecting the $BaCl_2$ solution on top of the gel. At a distance from the interface y >1.5 mm, the pH is constant, around 10.8.

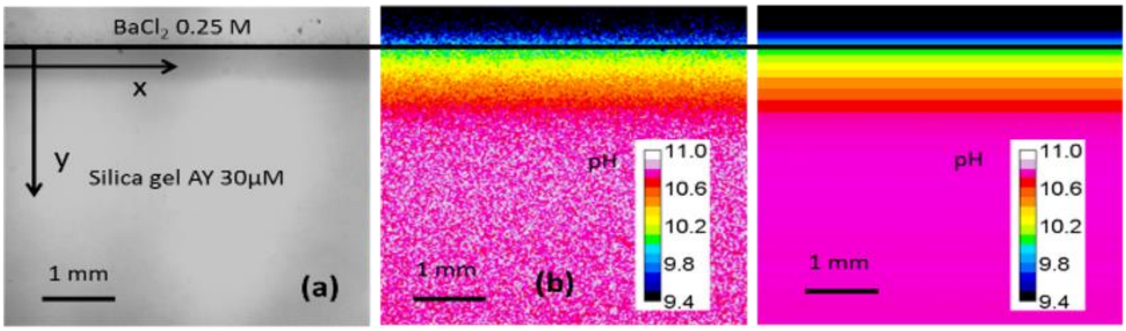

Figure S7. Optical transmission image and pH image of the silica gel. (a) Optical transmission image of the silica gel in the cassette taken 2 minutes after the injection of the $BaCl_2$ solution. The black line identifies the solution (top) –gel (bottom) interface. (b) pH image calculated from the ration R of fluorescence images F_1 and F_2 acquires upon excitation at 390 nm and 445 nm, respectively.

To calculate the standard deviation of the pH measured by ratiometric fluorescence imaging, the distribution of the pH in an area of 2x2 mm at distance y>1.5 mm from the interface was analyzed in the histogram shown in Figure S8a: the mean of the pH distribution, 10.8±0.05, matched very well the expected value.

pH profile across the x-axis parallel to the interface gel-solution for y values 0.5, 0.7, and 3.0 mm is shown in Figure S8b. Considering the homogeneous pH distribution observed in the x-direction, we average the pH in this direction to improve the signal-to-noise ratio in the pH detection in the y-direction. The resulting one-dimensional diffusion profile showing pH as a function of the position y is plotted in Figure S8c, which displays the pH's time evolution in the gel.

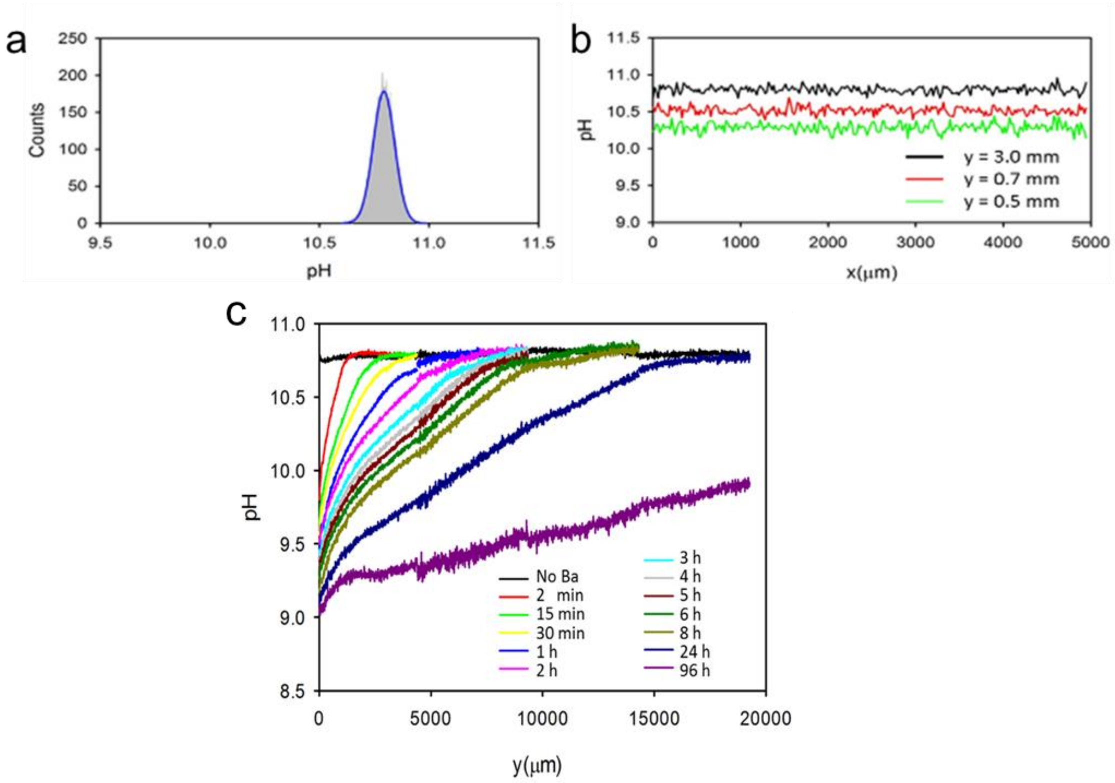

Figure S8. **Characterization of pH behavior in the silica gel.** (a) pH histogram in the silica gel at distance y>1.5 mm from the interface. The peak was fitted with a Gaussian profile to give pH=10.8±0.05. (b) pH profiles along the x-direction at different y positions (0.5, 0.7, and 3 mm). (c) pH profile along the y-direction as a function of time

Correlation between pH maps and crystal formation (Figure 3 a-i in the manuscript)

Transmission images were correlated with pH maps, demonstrating that no crystals are formed at the initial pH of the gel (10.8) but that a decrease in the pH associated with the $BaCl_2$ diffusion is necessary for the formation of detectable crystals. The highest pH required for the crystal formation at different times can be determined by identifying the crystal formed in the highest pH region, and hence the farthest ones from the interface (the line perpendicular to the y-direction corresponding to the position of these crystals is referred to a crystallization front). According to this approach, Figure 3b shows that the formation of a consistent number of crystals in the 0<y<0.5 mm section of the gel is observed after 15 min. The pH is now in that region of nucleation and growth as low as 9.5. After 15 min, the maximum pH compatible with crystallization is 10.4-10.5, and the crystallization front is positioned at y= 1.0 mm. It is interesting to observe that during the time, the crystallization front moves far from the interface (Figures 3c -3h), but the pH maintains the 10.4-10.5 value.

[1] S. G. Schulman, D. V. Naik, A. C. Capomacchia, T. Roy, *Journal of Pharmaceutical Sciences* **1975**, *64*, 982-986.
